# First helminthological data on the Iberian adder, *Vipera seoanei*

**DOI:** 10.1007/s00436-023-07849-9

**Published:** 2023-04-24

**Authors:** V. Roca, F. Gómez-Ramírez, I. Espasandín, R. Megía-Palma, A. Perera, F. Martínez-Freiría

**Affiliations:** 1 Departament de ZoologiaFacultat de Ciències Biològiques, Universitat de València, C/Dr. Moliner, 50, 46100 Burjassot, Spain; 2 Grupo de Investigación en Bioloxía Evolutiva (GIBE), Departamento de Bioloxía, Facultade de Ciencias, Universidade da Coruña, Campus da Zapateira, S/N, 15071 A Coruña, Spain; 3 CIBIO, Centro de Investigação Em Biodiversidade E Recursos GenéticosInBIO Laboratório Associado, Campus de Vairão, Universidade Do Porto, P-4485-661 Vairão, Portugal; 4 BIOPOLIS Program in Genomics, Biodiversity and Land Planning, CIBIO Campus de Vairão, P-4485-661 Vairão, Portugal; 5 Department of Biomedicine and Biotechnology, School of Pharmacy, Universidad de Alcalá (UAH), 28805 Alcalá de Henares, Madrid, Spain

**Keywords:** Helminths, Vipers, Ecology, Iberian Peninsula

## Abstract

The helminth fauna present in the gut contents of Iberian adders, *Vipera seoanei* (Squamata: Viperidae), were characterised and analysed in respect to biological and eco-geographic factors that may affect the occurrence and diversity of helminths in this species. A total of 317 samples of preserved stomachs and intestines, covering the distributional range of *V*. *seoanei*, were examined. Similar to other *Vipera* species from the Iberian Peninsula, the helminth fauna was also impoverished in *V*. *seoanei*, but unlike other *Vipera* species from Central and East Europe, helminths were mostly found in adult vipers, and occurred in vipers located at the periphery of the species range, characterised by low elevation, high temperature and precipitation levels, and abundant pastures.

## Introduction

The Iberian adder, *Vipera seoanei* (Lataste 1879), is a small-sized European viper (Fam. Viperidae, genus *Vipera*) clustered within the *Pelias* clade (Freitas et al. 2020). Nearly endemic to the northern Iberian Peninsula, *V*. *seoanei* is a typical predator of areas with Atlantic climate, frequently inhabiting humid environments of temperate forest, scrublands, pastures and agriculture fields, from sea level to 1900 m.asl in the Cantabrian Mountains (Martínez-Freiría and Brito 2014; Brito 2021).

Although the studies on the community ecology of parasites in European reptiles have increased in the last few decades (Martin and Roca 2005; Megía-Palma et al. 2018; Drechsler et al. 2021a, b; Dajčman et al. 2022), snake parasites have received limited attention (but see Aho 1990; Santos et al. 2006; Tomé et al. 2012, 2014). In this sense, two of the three Iberian viper species, *Vipera aspis* Linnaeus 1758 and *Vipera latastei* Boscá 1878, have been helminthologically studied (Sánchez-Mut et al. 2004; Santos et al. 2006; Ribas et al. 2010). However, to date, there is no parasitological data for *V*. *seoanei*.

In this work, we study the helminth fauna present in the gut contents of *V*. *seoanei*. Considering the low prevalence of intestinal helminths in the already studied Iberian viper species (Santos et al. 2006), we predict a poor gastrointestinal helminth community for *V*. *seoanei*. This pattern may be explained by the trophic ecology of Iberian vipers, which is characterized by long periods without eating and a rather specialised diet, based on small mammals in adult vipers, and amphibians and lizards in immature ones (Santos et al. 2006; Espasandín et al. 2022). Yet, other European vipers such as *Vipera berus* (Linnaeus, 1758), a sister taxa to *V*. *seoanei*, which is distributed across northern Europe and also linked to humid environments (Martínez-Freiría et al. 2015; Freitas et al. 2020), show richer and more diverse helminth communities (Shimalov and Shimalov 2000; Ribas et al. 2010; Kusmierek et al. 2020; Kirillov and Kirillova 2021). Consequently, we expect that the occurrence of helminths in *V*. *seoanei* could correlate with humid environments as depicted by eco-geographic factors. We also expect that adults will be more infected than non-adults, simply because of the greater chance of infection in older individuals (e.g. Roca et al. 1990).

By presenting the first helminthological data for *V*. *seoanei*, we tend to address the following issues: (i) to characterise the patterns of helminth infection and diversity; and (ii) to analyse the biological and eco-geographic factors that could relate to the occurrence of parasites in this species. By doing so, we also compare the helminthological data from this host with those from other Iberian and European vipers.

## Materials and methods

### The data

**Fig. 1:**
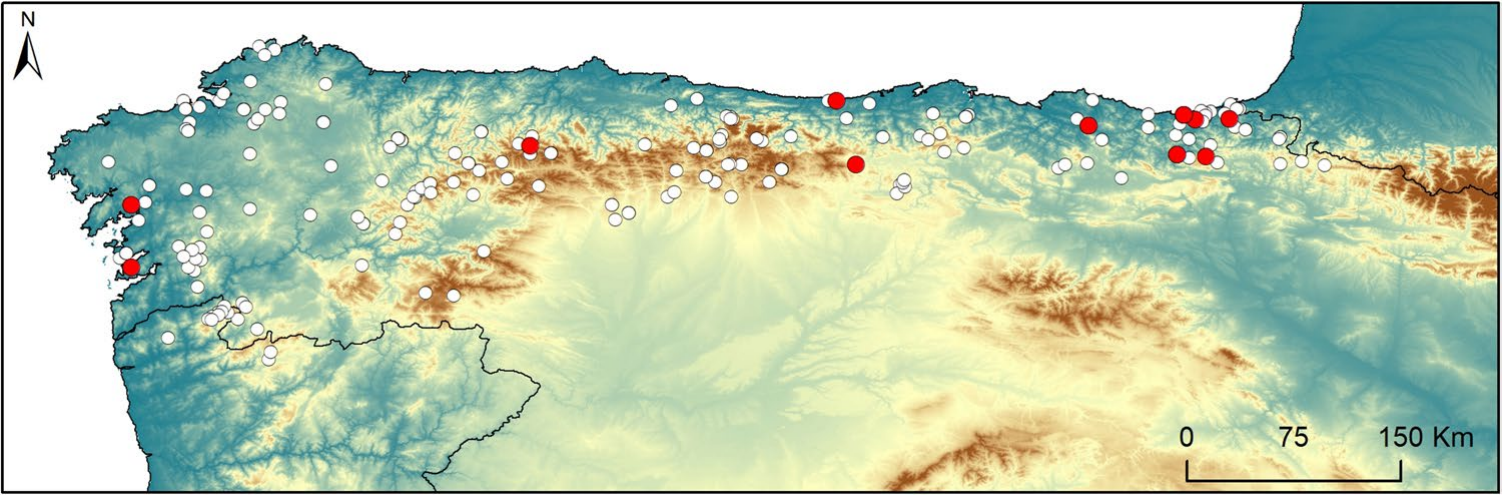
Distribution of the specimens examined in this study (white dots), also depicting the location of vipers with helminths (red dots)

We analysed samples from 317 specimens (Table 1), mostly covering the distribution range of *V*. *seoanei* (Fig. 1). Samples consisted of stomachs and intestines preserved in 70% ethanol, resulting from a previous dissection performed to study the species feeding ecology (Espasandín et al. 2022). The dataset included samples from (i) ethanol-preserved specimens stored in three Spanish collections, Museo Nacional de Ciencias Naturales—CSIC, Madrid (*n* = 163; collected from 1968 to 2011), Sociedad de Ciencias Aranzadi, Donostia (*n* = 76; collected from 1974 to 2013) and Departamento de Biología, A Coruña University, A Coruña (*n* = 22; collected from 1976 to 2010); and (ii) ethanol-preserved roadkill specimens from the particular collection of F. Martínez-Freiría (*n* = 43; collected from 2004 to 2021), and J.C. Brito (*n* = 13; collected from 1996 to 2008) (CIBIO, Vairao, Portugal). Only the roadkill specimens that were in good condition (in which the digestive tract was practically intact, and which were preserved soon after the road accident) were analysed for helminths.

**Table 1:** Number of analysed samples, parasitized vipers and prevalence of infection

	Analysed	Parasitized	Prevalence
Males	157	10	6.4%
Females	99	5	5.1%
Indeterminated sex	61	0	0%
Adults	199	14	7%
Non adults^a^	118	1	0.8%
Total	317	15	4.7%

^a^Subadults and juveniles, SVL < 325 mm (sensu Espasandín et al. 2020)

### Parasites determination

All the samples were examined under stereoscopic microscope. The helminths found were removed, washed, fixed and mounted according to standard techniques (Galdón 2007). All the parasites were identified to the species level when possible (see Yamaguti 1961; Hartwich 1974; Lichtenfels 1978). Infection parameters described by Bush et al. (1997) were used to define the ecology of the parasites. Thus, prevalence is the number of infected hosts divided by the total number of individual analysed; intensity of infection is the total number of parasites divided by the total number of infected hosts; abundance is the total number of parasites divided by the total number of individual analysed (Rózsa et al. 2000).

### Biological and eco-geographic predictors

We consider two sets of predictors that could affect the occurrence of parasites in vipers. These two sets of predictors were previously considered in the study of the feeding activity of *V*. *seoanei* (Espasandín et al. 2022) and frequently relate to the ecology of the Iberian viper species (e.g. Martínez-Freiría et al. 2008, 2015; Lucchini et al. 2020; Chamorro et al. 2021). Further information on how they were obtained can be found in Espasandín et al. (2022).

The first set comprises of three biological traits, intrinsic to each viper: SEX (categorical variable, two categories: female/male), SVL (Snout-Vent Length, continuous variable, measured in mm) and PREY (categorical variable with two categories: presence/absence of prey in the gut). The second set consists of nine continuous eco-geographic predictors that describe the environmental conditions of the geographic position where each viper was collected. It includes (i) five topo-climatic factors, ELEV (elevation about sea level, in m), Mean T (annual mean temperature, in °C), Max T (maximum temperature of the warmest month, in °C), APrec (annual precipitation, in mm) and DrPrec (precipitation of the driest month, in mm); and (ii) four habitat types, expressed as percentages of FOREST (forest), PAST (pastures and grasslands), MOORS (moors and heathlands) and AGRIC (agricultural areas).

### Statistical analyses

To address the univariate relation between the occurrence of parasites and biological and eco-geographic predictors, we performed two types of statistical tests: (i) Fisher tests in the case of categorical biological variables (i.e. SEX and PREY); and (ii) univariate logistic regressions in the case of continuous biological (i.e. SVL) and eco-geographic variables (i.e. ELEV, MeanT, MaxT, APrec, DrPrec, FOREST, PAST, MOORS and AGRIC). In the latter, the significance of the predictor coefficients (ßs) was evaluated by maximum likelihood χ2 tests. Significant predictors were plotted to further understanding their influence. Statistical analyses were performed in R. Studio version 1.1.463, using the available family of GLM stats in R (R Core Team 2022), and the package “effects” to plot predictor’s responses.

## Results

**Fig. 2:**
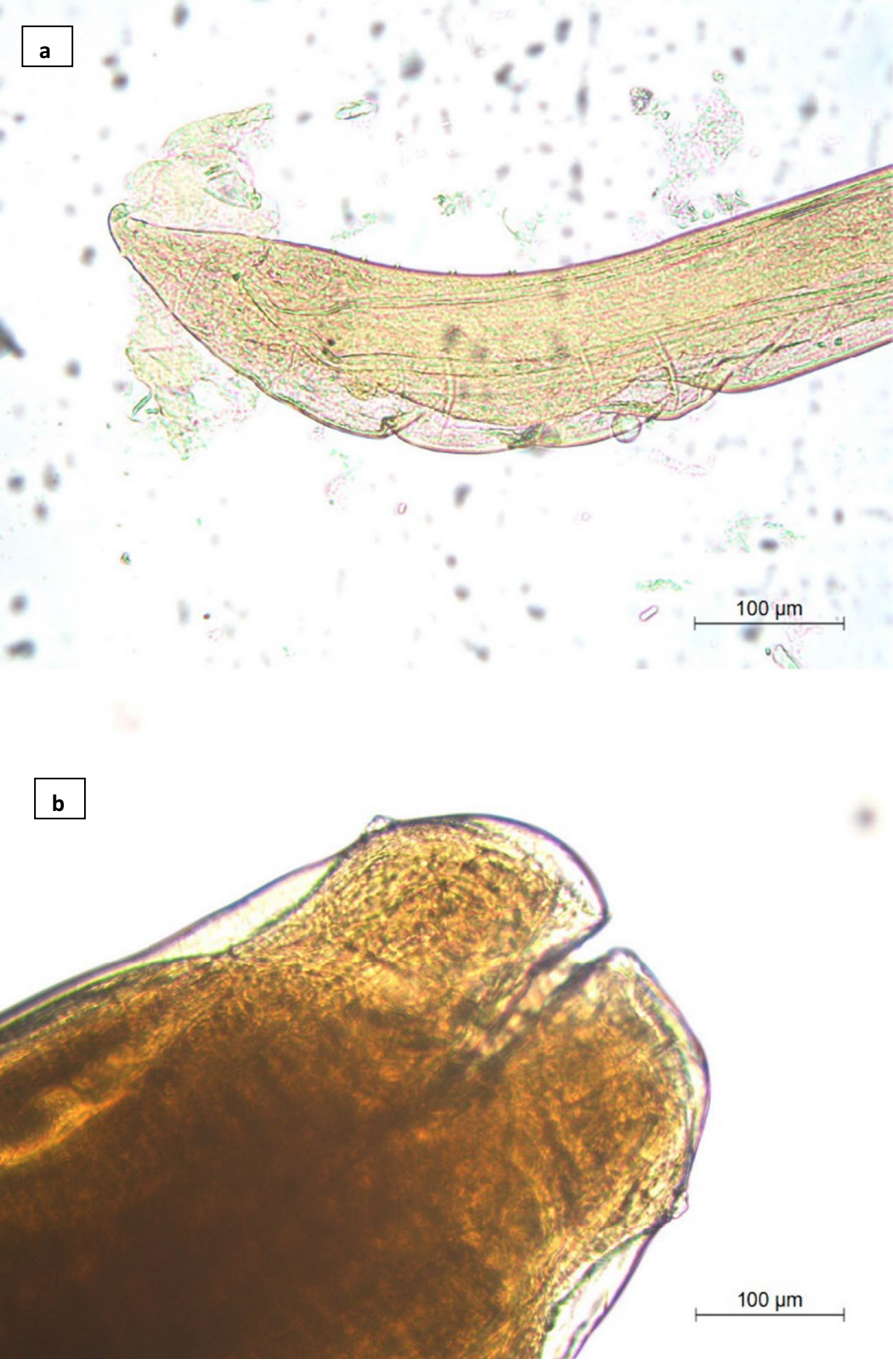
**a** *Oxysomatium brevicaudatum*, caudal end of the male in ventral view; **b** *Ophidascaris* sp., anterior end of female in lateral view

**Fig. 3:**
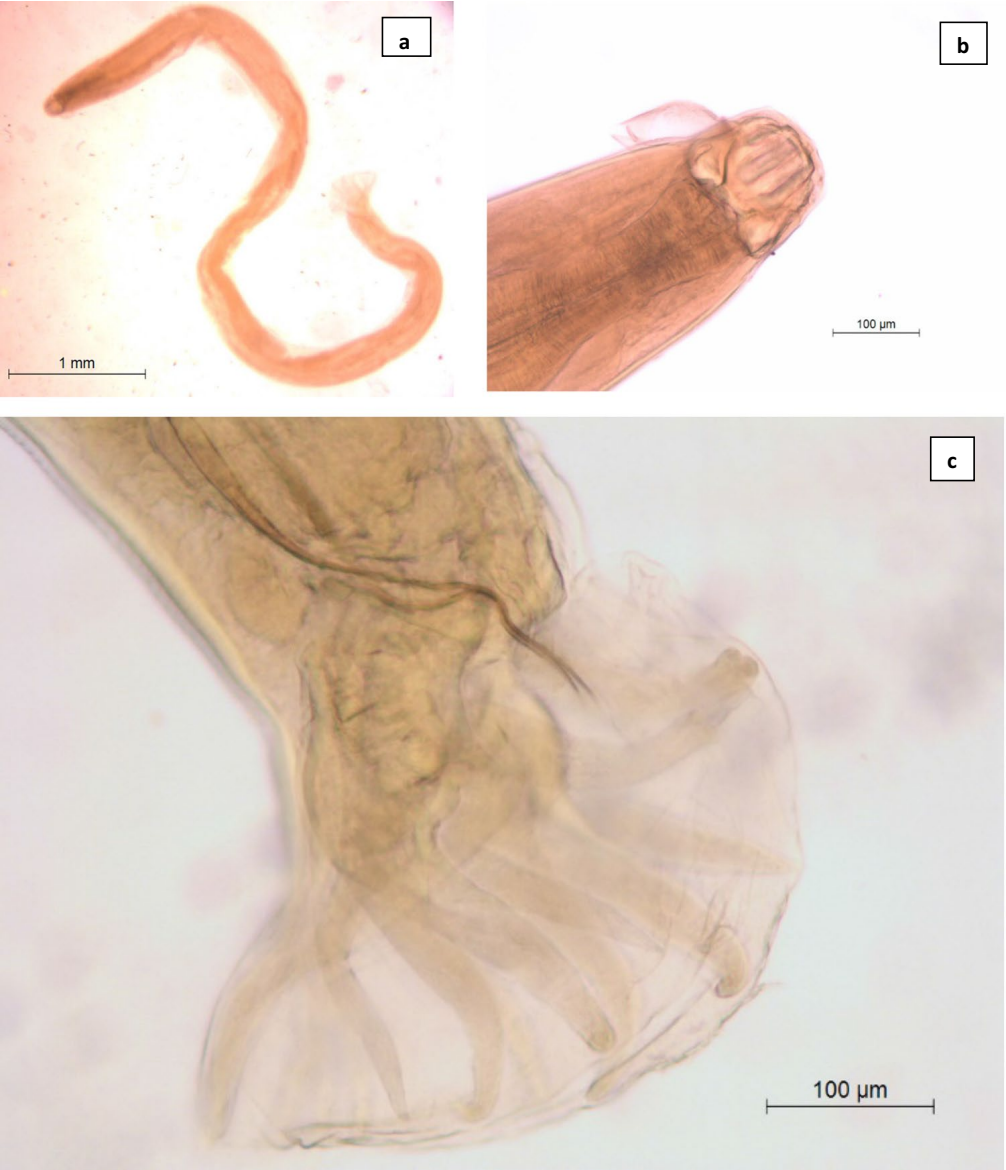
*Kalicephalus viperae*. **a** Male; **b** cephalic end of male in ventral view; **c** caudal bursa of male in ventral view

Three helminth species (all nematodes) were found parasitizing *V*. *seoanei* (Table 2): *Oxysomatium brevicaudatum* (Schneider 1866) (Fig. 2a), *Kalicephalus viperae* (Rudolphi 1819) (Fig. 3) and *Ophidascaris* sp. (Fig. 2b). All of them were found at the anterior part of the intestine. The prevalence of infection was 4.7% (i.e. 15 out of 317 specimens, Table 1); the mean (± standard deviation) intensity was 5.8 ± 10 (1–38) and the mean abundance was 0.3 ± 2.4 (0–38). The mean values of parasitism diversity parameters for *V*. *seoanei* were not calculated due the very poor helminth infracommunities found.

**Table 2:** Infection parameters of helminth species parasitizing *V*. *seoanei* from Iberian Peninsula

Helminth species	Prevalence	Mean intensity	Mean abundance
*Oxysomatium brevicaudatum*	0.6%	–	–
*Kalicephalus viperae*	0.6%	21.5 ± 23^a^ (5–38)^b^	0.1 ± 2.2 (0–38)
*Ophidascaris* sp	3.5%	3.8 ± 5.4 (1–19)	0.1 ± 1.2 (0–19)

^a^Standard deviation, ^b^Range

**Fig. 4:**
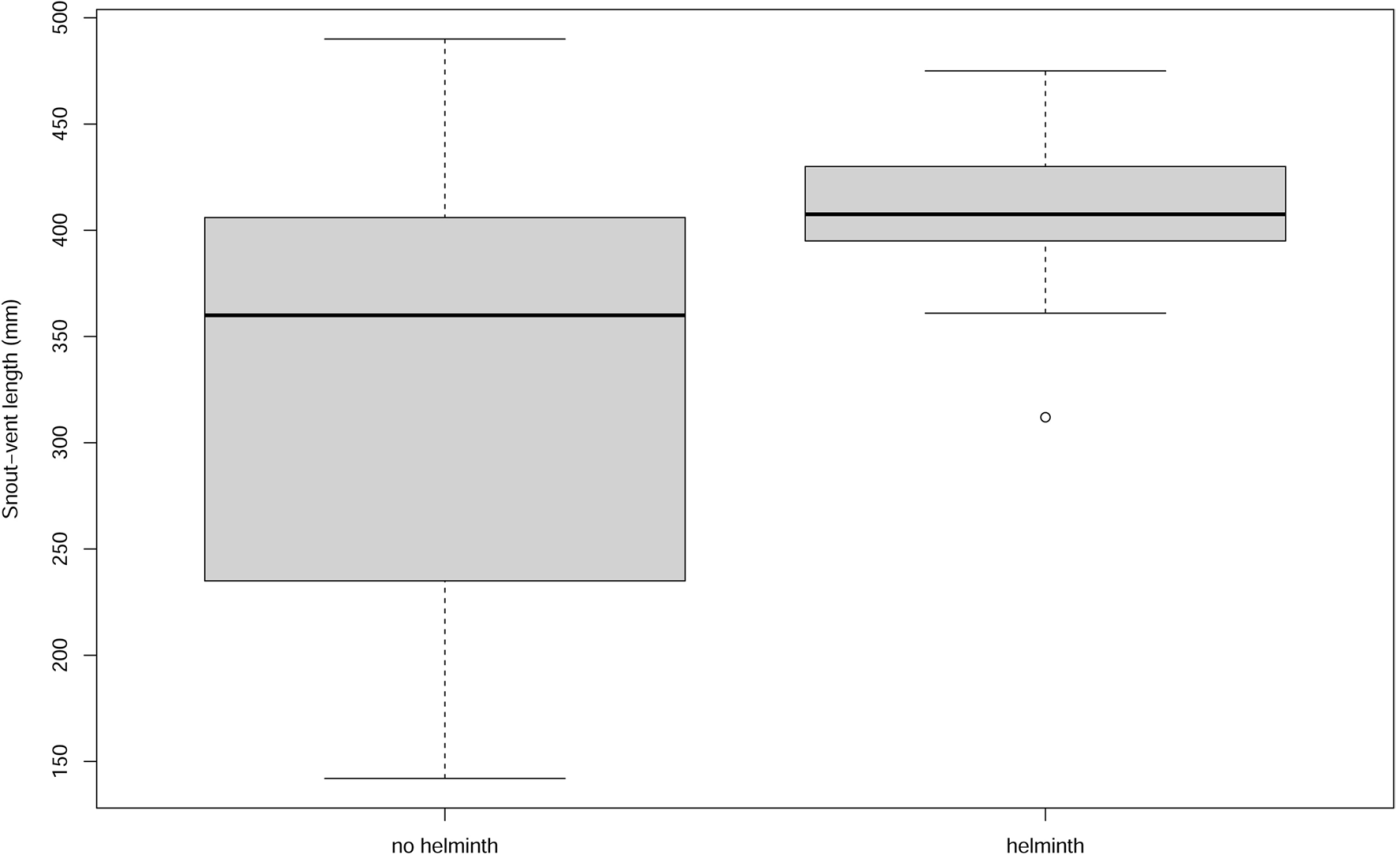
Occurrence of vipers with and without helminths in relation to SVL

Helminths were mostly found in adult vipers (Table 1), showing a significant effect of SVL on their occurrence (Table 3; Fig. 4). Although helminths were more frequently found in males than in females (Table 1), this difference was not statistically significant (Fisher test, *p* = 0.787). Helminths were also found in similar proportion in vipers with (*n* = 8) and without (*n* = 7) prey in the guts (Fisher test, *p* = 0.173).

**Table 3:** Maximum Likelihood χ2 tests to evaluate the significance of the predictor coefficients in the univariate logistic regressions for the occurrence of parasites in *V. seoanei*. Significant predictors (*P* < 0.05) are depicted in bold

	Df	Deviance Resid	Df	Resid. Dev	Pr (> χ2)
**SVL**	297	112.96			
	1	12.275	296	100.680	< 0.001
**ELEV**	295	118.7			
	1	12.383	294	106.31	< 0.001
**MeanT**	295	118.7			
	1	9.7219	294	108.970	0.002
MaxT	295	118.7			
	1	0.67041	294	118.030	0.413
APrec	295	118.7			
	1	0.046177	294	118.650	0.830
**DrPrec**	295	118.7			
	1	13.993	294	104.700	< 0.001
FOREST	295	118.7			
	1	1.2117	294	117.480	0.271
**PAST**	295	118.7			
	1	4.6639	294	114.030	0.031
MOORS	295	118.7			
	1	0.69461	294	118.000	0.405
AGRIC	295	118.7			
	1	2.9092	294	115.790	0.08807

**Fig. 5:**
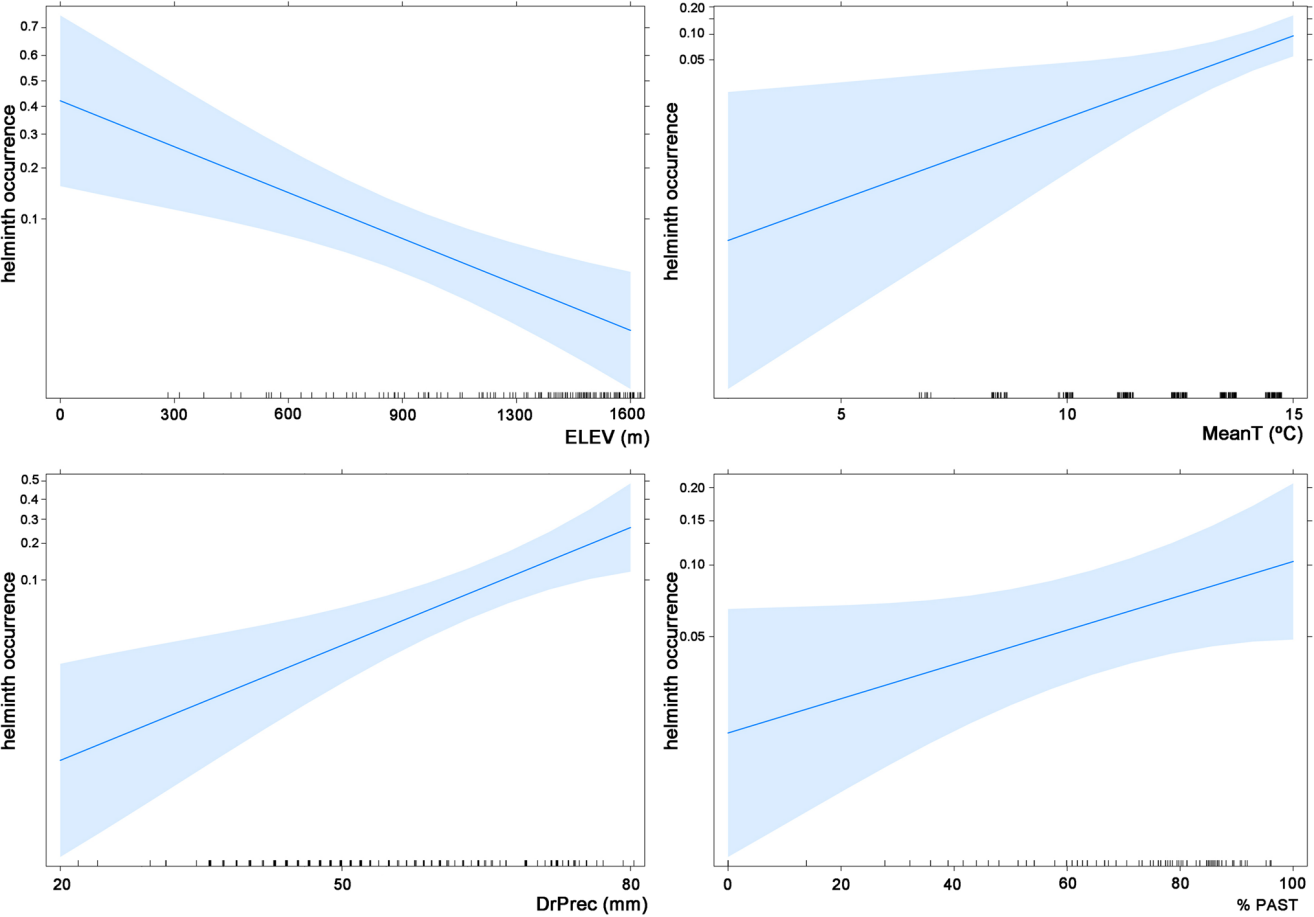
Response plots for the logistic regressions of the occurrence of helminth and three topo-climatic (ELEV, MeanT and DrPrec) and one habitat (PAST) factors. Filled zone represent 95% of confidence interval

The occurrence of helminths was significantly related to ELEV, MeanT, DrPrec and PAST (Table 3). Helminths occurred in vipers located at the periphery of the species range (Fig. 1), characterised by the low elevation, high temperature and precipitation levels, and abundant pastures (Fig. 5).

## Discussion

### Patterns of helminth infection and diversity

*Oxysomatium brevicaudatum* (Schneider 1866) (fam. Cosmocercidae) (Fig. 2a) is a nematode usually found in several species of amphibians and water snakes from Europe (Lewin 1992). Lewin and Grabda-Kazubska (1997) point out that vipers represent postcyclic hosts for this nematode species, i.e. vipers ingest already infected prey hosts (Bozhkov 1969). The low prevalence and number found of this helminth conform the findings of previous studies (Lewin and Grabda-Kazubska 1997).

The second species found, *Kalicephalus viperae* (Rudolphi, 1819) (fam. Diaphanocephalidae) (Fig. 3a), is a hookworm that has previously been found in a wide spectrum of colubrid and viperid hosts from Europe (Ribas et al. 2010). It shows a characteristic buccal capsule surrounded by three lips at each side (Fig. 3b), and males show a copulatory bursa (Fig. 3c) that is typical of strongyloids (Lewin 1993). Its infection may cause anaemia, haemorrhagic ulcers and intestinal obstruction (Barnard 1986).

The third species found was *Ophidascaris* sp. The most likely centre of dispersal for the genus *Ophidascaris* (fam. Ascarididae) would be central Africa with posterior spreads into Asia and into Madagascar and Australia (Sprent 1988). Although the only species of this genus cited in Europe is *O*. *natricis* (in Russia, Bogdanov 1954), finding only slight differences in body measurements between our specimens and the description of the species (Yamaguti 1961), makes us leaving species assignment as *Ophidascaris* sp (Fig. 2b). This genus has been cited parasitizing vipers and other snakes in Spain where it seems common (Ribas et al. 2010). However, it has not been found in other parts of Europe (Lewin 1992, 1993; Lewin and Grabda-Kazubska 1997).

*Ophidascaris* sp. and *K*. *viperae* can be considered as host specialists (Edwards and Bush 1989) since they only parasitize snakes (Yorke and Maplestone 1969; Sharpilo 1976). However, the very low values of prevalence and mean intensity and abundance of infection reported in our study (Table 2) suggest that these helminths occur occasionally in *V*. *seoanei*. Our results agree with those of *V*. *aspis* and *V*. *latastei* from Spain (Santos et al. 2006; Ribas et al. 2010) and with data from other Iberian snakes (Roca et al. 2021). Interestingly, they contrast with data on European and American vipers that exhibit wide and diverse heminth faunas (Santos et al. 2006; Kusmierek et al. 2020). These discrepancies between the Iberian and other European viper hosts suggest an influence of the paleo-tectonic and climatic history of the Iberian Peninsula in shaping (and limiting) the infracommunities of Iberian vipers, as has been already indicated for the biogeographic history of these viper hosts (Martínez-Freiría et al. 2015, 2020), as well as other snake hosts (e.g. Santos et al. 2008).

### Biological and eco-geographic factors related to helminth occurrence

Body length (measured as SVL) was the single biological trait showing a significant effect on parasite prevalence (Table 3; Fig. 4). Assuming that sexual maturation occurs at 325 mm of SVL (Lucchini et al. 2020; Espasandín et al. 2022), we indicate that helminths were mostly found in adults of *V*. *seoanei*. This agrees with results from other reptile species in which body length also has a significant effect on the occurrence of parasites (e.g. in lacertid lizards, Roca et al 1990; Martin and Roca 2004; Roca et al. 2006). Furthermore, this seems to be a common pattern among short-lived reptile species infected by blood parasites (Maia et al. 2014). Nevertheless, in the snake host *Natrix natrix* (Linnaeus 1758) (fam. Natricidae), certain parasites are found in larger individuals, while others are increasing infection in smaller snakes (Lewin 1992). The possible reasons for greater parasitisation of adults *versus* non-adults could be mediated by (i) a greater chance of infection due to longer lifespan and thus, time of parasite recruitment, and/or (ii) increased possibilities of contact with other adult vipers due to sexual behaviour (e.g. during copulation, territorial fights) (Roca et al. 1990; Maia et al. 2014).

A major prevalence of intestinal parasites in females of *V*. *seoanei* could be expected due to the higher feeding frequency (Espasandín et al. 2022), which could lead to increasing opportunities of parasite infection (e.g. Norris 1999; Roca et al. 2005; Carretero et al. 2014). However, our results indicate that sex had no influence on *V*. *seoanei* parasite prevalence. This result is consistent with previous observations in other European snake and lizard hosts in which there were no significant differences in the prevalence of intestinal parasites between females and males (Roca et al. 1990; Lewin 1992; Lewin 1993; Martin and Roca 2005). Conversely, it contrasts with other groups of parasites that infect reptiles, such as ectoparasites or blood parasites, in which males were infected with greater frequency and/or intensity (Amo et al. 2005; Álvarez-Ruiz et al. 2018; Drechsler et al. 2021a, b). This fact has often been explained by the combined effects of immunosuppressive cause of testosterone and a higher movement rate in the males of some host species (Olsson et al. 2000; Wieczorek et al. 2020; Barrientos and Megía-Palma 2021).

Parasite prevalence can be influenced by distinct factors such as environmental conditions (Carbayo et al. 2019; Megía-Palma et al. 2020; Rivera-Rea et al. 2022). We found a positive correlation between the occurrence of helminths and three topo-climatic (ELEV, Mean T and DrPrec) and one habitat (PAST) predictor (Table 3). Likewise in other reptile hosts, helminth infection in vipers can be strongly influenced by the selection of microhabitats and the prey items they consume (Brito et al. 2014). For example, snake species living in dry habitats and feeding on rodents are weakly infected by helminths (Kirillov and Kirillova 2021). Although the factors found here as predictors of helminth occurrence are not the same as those related to the consumption of major prey types in this viper species (Espasandín et al. 2022), they align with the aforementioned pattern in *V*. *seoanei*. Helminths occur in vipers inhabiting lowlands, areas of high temperature and precipitation levels, and with abundance of pastures (Fig. 5). These environments relate to occurrence of amphibians (Sillero et al. 2009), which are common prey of *V*. *seoanei* (Espasandín et al. 2022) and, at the same time, important hosts for a rich infracommunity (Sánchez 1998).

Sharpilo (1976) and Lewin and Grabda-Kazubska (1997) pointed out that there are no host-specific parasites for *V*. *berus.* Accordingly, many parasite species are adopted from water snakes (genus *Natrix*) in localities where populations of both types of snakes coexist. In this sense, the very low number of parasite species found in *V*. *seoanei* suggests the non-coexistence of vipers and water snakes in the same localities. A fact that reinforced our observations gathered in the field (authors, personal observations).

Undoubtedly, these are the first insights into the helminth infracommunity of *V*. *seoanei* and many questions remain unaddressed due to the low parasite prevalence detected in this host species. Future studies should focus on studying helminth fauna with complementary methods that add diagnose sensitivity to the visual inspection of specimens stored in collections (e.g. metabarcoding, Bourret et al. 2021).

## Data Availability

The data and parasitological material are available in the Department of Zoology, Faculty of Biological Sciences, University of Valencia.
